# Preparation and characteristic analysis of nanofacula array

**DOI:** 10.1038/s41598-021-01637-0

**Published:** 2021-11-12

**Authors:** Lina Shao, Xin Tian, Shengxiang Ji, Hongda Wang, Yan Shi

**Affiliations:** 1 State Key Laboratory of Electroanalytical Chemistry, Changchun Institute of Applied Chemistry, Chinese Academy of Sciences, Changchun, 130022 Jilin China; 2 State Key Laboratory of Applied Optics, Changchun Institute of Optics, Fine Mechanics and Physics, Chinese Academy of Sciences, Changchun, 130022 Jilin China; 3 University of Science and Technology of China, Hefei, 230026 China; 4 Key Laboratory of Polymer Ecomaterials, Changchun Institute of Applied Chemistry, Chinese Academy of Sciences, Changchun, 130022 Jilin China; 5 Laboratory for Marine Biology and Biotechnology, Qingdao National Laboratory for Marine Science and Technology, Qingdao, 266200 Shandong China

**Keywords:** Engineering, Optics and photonics

## Abstract

The development of nanofacula array is an effective methods to improve the performance of Near-field Scanning Optical Microscopy (NSOM) and achieve high-throughput array scanning. The nanofacula array is realized by preparing metal nanopore array through the "two etching-one development" method of double-layer resists and the negative lift-off process after metal film coating. The shading property of metal film plays important rules in nanofacula array fabrication. We investigate the shading coefficient of three kinds of metal films (gold–palladium alloy (Au/Pd), platinum (Pt), chromium (Cr)) under different coating times, and 3.5 min Au/Pd film is determined as the candidate of the nanofacula array fabrication for its lower thickness (about 23 nm) and higher shading coefficient (≥ 90%). The nanofacula array is obtained by irradiating with white light (central wavelength of 500 nm) through the metal nanopore array (250/450 nm pore diameter, 2 μm pore spacing and 7 μm group spacing). Moreover, the finite difference and time domain (FDTD) simulation proves that the combination of nanopore array and microlens array achieves high-energy focused nanofacula array, which shows a 3.2 times enhancement of electric field. It provides a new idea for NSOM to realize fast super-resolution focusing facula array.

## Introduction

In 1928, British scientist Synge put forward an assumption that can break through the resolution of optical microscope for the first time. Synge suggested that super-resolution imaging could be achieved by scanning and collecting the light signal of sample point by point with a detector of sub-wavelength size placed in the near-field range of the sample, which is the origin of near-field scanning optical microscope (NSOM)^1^. The classical NSOM provides a sub-wavelength, near-field light source by the utilization of an aperture tip to limit the incident light to the nanometer scale. By collecting the near field evanescent wave scattered into the far field, NSOM can simultaneously perform high-resolution fluorescence and topography images^2^. NSOM breaks through the optical diffraction limit and achieves unprecedented resolution^3^. Also, it retains the unusual characteristics of optical microscope, such as non-invasion, stability, low cost, as well as the optical contrast mechanism similar to traditional optics, which gives NSOM considerable versatility^4,5^. However, the low time resolution is the key factor that limit the development of NSOM in the biologic research, because of that long scanning time is required for large area and high resolution imaging, which cannot meet the study of single molecule localization and dynamic process in cells^6^.

The research and development of nanofacula array can not only meet the limit of incident light, but also achieve the transformation from a single to an array light source, which improves the time resolution of NSOM. The nanofacula array is realized by preparing the metal nanopore array with regular shape and controllable size^7^, which uses double-layer resist exposure and negative lift-off process after metal film coating. Electron beam etching (EBL) is one of the most widely used nano-lithography technology, which is applied to fabricate templates in the semiconductor industry and other nano-manufacturing fields^8^. The most commonly used EBL resists include hydrosesquisiloxane (HSQ) and polymethyl methacrylate (PMMA), which have excellent photolithographic output and processability. HSQ is a high-resolution negative resist, which belongs to the polysiloxane family of inorganic materials^9^. For salt developer, HSQ can be used to prepare nanostructures with high contrast and resolution below 10 nm^10^. PMMA is commonly considered as a positive resist with appropriate sensitivity, high contrast and low roughness. The high-resolution structure with PMMA can be achieved by using high beam voltage, low temperature and high beam dose^11^. For the preparation of metal structures protruding on the substrate, positive resist is usually applied in EBL; whereas, it is necessary to fabricate sunken metal structure using PMMA-HSQ double-layer resist. With this method, the Rommel’s group made a square metal block array of side-length 100 nm and a spacing less than 10 nm in a large range of 100 μm × 100 μm^12^. In addition, metal wires^13^, metal holes^7^, metal gratings^14^ and metal concentric circles^15^ of various sizes are also prepared.

In general, the method of coating metal or semiconductor materials contains physical methods (thermal evaporation, sputtering) and chemical methods (vapor deposition, electroplating), in which sputtering method applies high-energy particles to attack the target and separate the atoms from the target in vacuum. This method avoids the resist pattern damage caused by high temperature and chemical reagents, and is suitable for coating materials on resist surface. In this work, we optimized the shading coefficient and coating time of different metal films, as well as the thickness of the PMMA according to the thickness of the metal film for development of a metal nanopore array applied to improve the performance of NSOM. Therefore, the good transmittance of these arrays of white light centered around 500 nm ensures the realization of nanofacula array, which advances the development of NSOM from single light source to array scanning. Additionally, an idea of combining nanopore array with microlens array is proposed in order to realize super-resolution focused facula array. It provides a new way for NSOM to realize super-resolution focused facula array.

## Methods

### Instruments and reagents

Positive resist: polymethyl methacrylate (PMMA, MICRO-CHEM, UK), molecular weight 495 K; negative resist: hydrosesquisiloxane (HSQ, Dow Corning, USA), model XR-1541-002; developer: 25% tetramethylammonium hydroxide, (TMAH, Sigma-Aldrich, USA); degumming agent: N-methyl-2-pyrrolidone (NMP, Sigma-Aldrich, America). Electron beam lithography and sample characterization are carried out on ultra-high resolution field emission scanning electron microscope (SEM, Zeiss, DEU); oxygen plasma etching is performed on PE50 plasma surface treatment machine (Plasma etch company, USA); metal films with different thickness is coated on high precision ion beam coating machine (GATAN, USA), and the height and thickness of samples are characterized on atomic force microscope (AFM-ICON, Bruker, DEU).

### Experimental methods

#### Thickness and shading coefficient of metal films

Gold–palladium alloy (Au/Pd), platinum (Pt) and chromium (Cr) are coated on the clean glass surface at different coating times of 1, 2, 4, 8, 16 min. In order to measure the thickness of metal films, tape is applied on part of the glass sheet. After metal coating, the tape is removed, and the thickness of the metal film is measured at the step by AFM. Scratch method is also used to measure the thickness of metal films (Supplementary Fig. S1). The shading coefficients of metal films are measured with power meter by recording the powers of laser passing through clean glass slide (P_g_) and metal coating samples (P_s_), and is defined as (P_g_ − P_s_)/P_g_. As the lift-off process requires that the thickness of the PMMA is at least twice of the metal film, which directly affects the stability of the mushroom structure, so it is necessary to select the thinnest metal film with the highest shading coefficient. For the Au/Pd film, the coating time is 8 min for the shading coefficient of 99%, but the thickness is more than 50 nm. It’s not suitable to use in the following lift-off process, so we choose the shading coefficient of 90% for both the good shading effect and the less metal film thickness, as detailed in Fig. 3a.

#### Preparation of metal nanopore array

**Figure 1:**
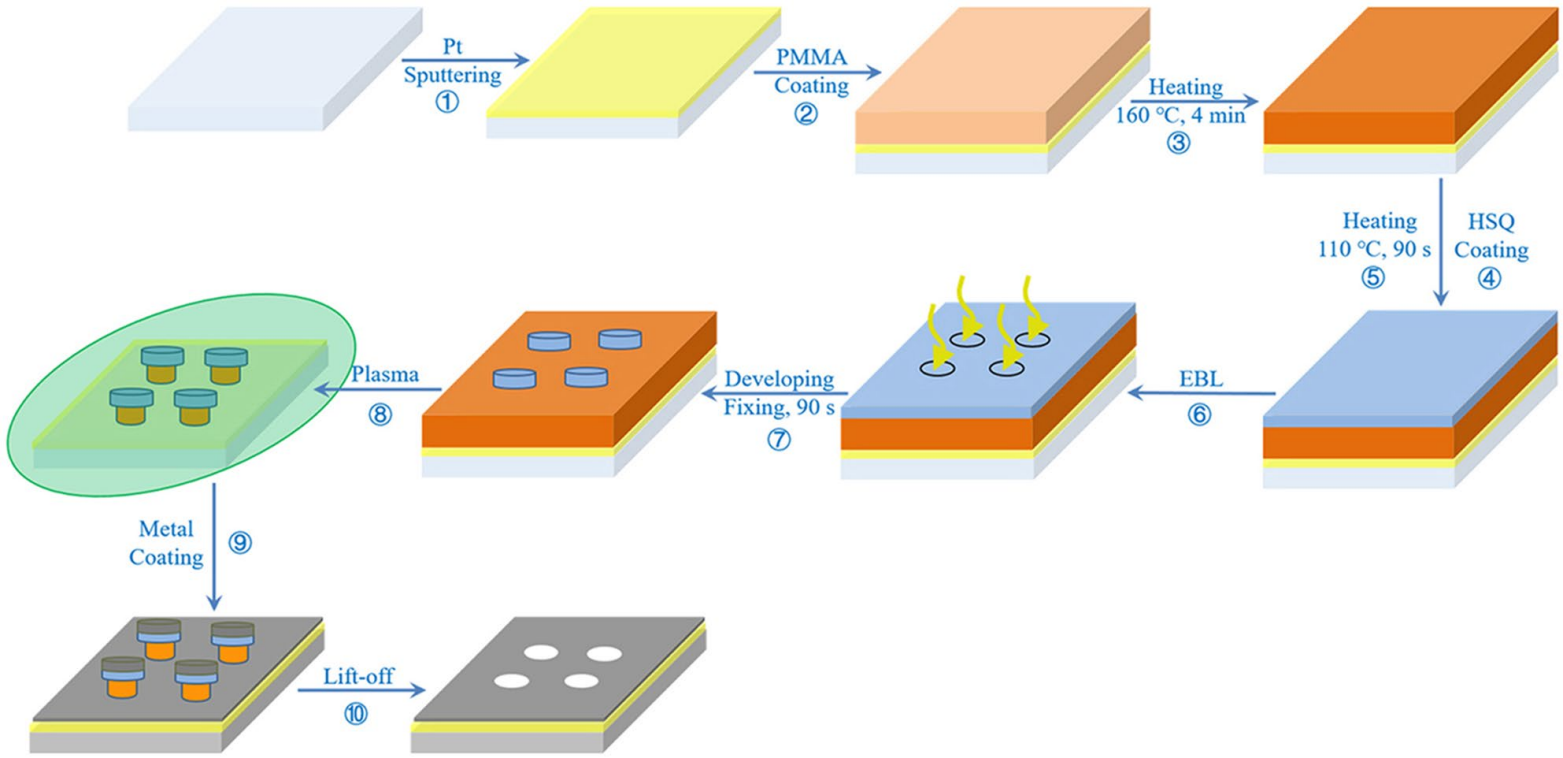
Synthesis process of metal nanopore array fabrication.

As shown in Fig. 1, the preparation process of the metal nanopore array is specifically described as follows: firstly, a very thin layer of Pt is sputtering on the surface of glass substrate to ensure both the conductivity and light transmittance of the substrate (①). According to the optimized thickness and spin-coating speed, PMMA and HSQ are spin-coated on the Pt-glass substrate. After each coating, the sample should be heated to avoid the fusion of double-layer resist (②–⑤). The pattern is realized through the EBL expose (⑥), development and fixing of HSQ (⑦), and the PMMA etching by plasma (⑧). The mushroom structure of HSQ capping-PMMA column is formed in the way of “two etching-one development” of the two resist. Subsequently, the Au/Pd atoms fall vertically to form a metal film by ion beam sputtering (⑨), and then PMMA column is lifted-off in the degumming solution, exposing the nanopore array without metal film (⑩) (details in supplementary).

#### Numerical simulation process of FDTD method

**Figure 2:**
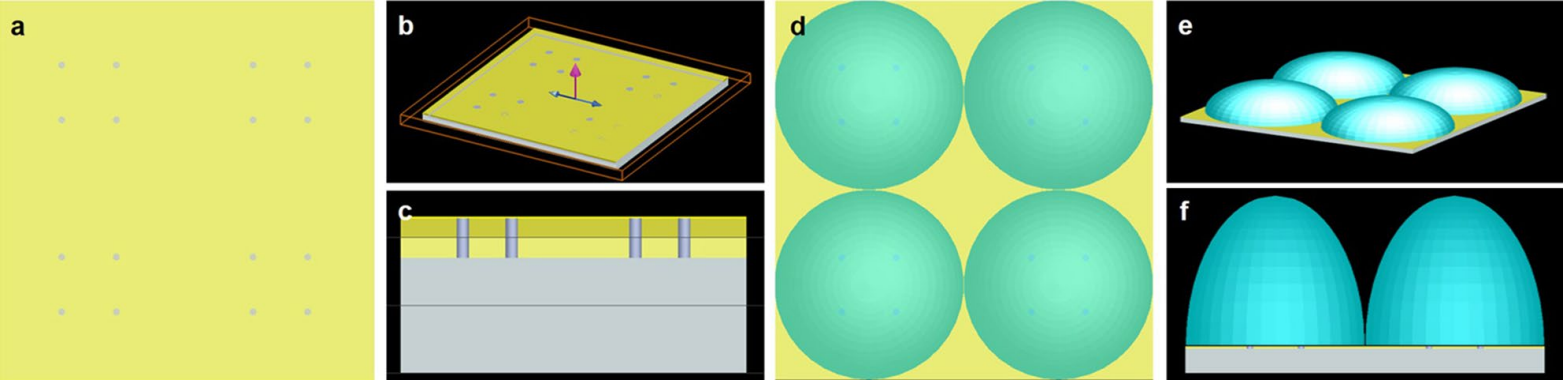
Numerical analysis model of nanopore array (**a**–**c**); Numerical analysis model of nanopore-microlens array (**d**–**f**).

The spectral data of the nanopore array can be obtained by using high numerical aperture (NA) microscope and spectrometer^16^. Though we tried many methods, the high light energy lose through various optical elements was the key limitation to obtain the experimental data. So we used the finite difference and time domain (FDTD) method^17^ to simulate and analyze whether the designed sub-wavelength periodic array structure has transmission enhancement effect at a certain wavelength. The model in the FDTD (Fig. 2a–c) was set as follows: four nanopores form a nanopore array unit, and the nanopore units form a periodic array structure, which are all etched through the metal film coated on glass substrate. The incident light is a plane wave with amplitude of 1 which propagates along the positive Z direction. The simulation parameters are set as follows: metallic material of gold (Au (gold)–CRC), gold film thickness of 60 nm, the diameter of the nanopore of 250 nm, the spacing between adjacent nanopores of 2 μm, and the spacing between nanopore array unit of 7 μm. As the FDTD material library does not contain Au/Pd, we use 60 nm Au film to replace 25 nm Au/Pd, for that their shading coefficients are similar in our experiment (data not shown). In our FDTD, three-dimensional (3D) calculations, simulation time and mesh size are 1000 fs and Δx = Δy = Δz = 25 nm, respectively. The perfectly matched layer boundary condition was applied at the grid boundaries (details in supplementary).

The nanopore array structure described above is combining with microlens array, and each nanopore array unit corresponds to a microlens (Fig. 2d–f). The optimized parameters of the microlens are as follows: establish four microlens whose center coordinates are x =  ± 3.5, y =  ± 3.5, the refractive index is 1.51, the radius of curvature is 3.5 μm, and the diameter of the microlens is 7 μm.

## Results and discussion

### Optimization of shading coefficient and thickness of metal films

**Figure 3:**
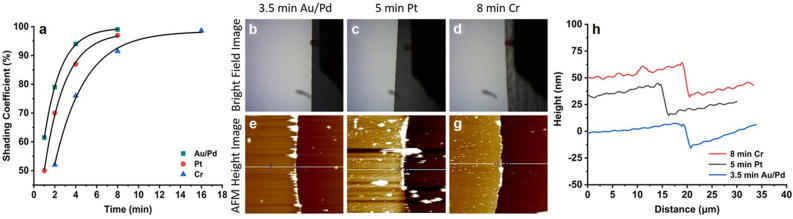
(**a**) Shading coefficient of three kinds of metals (Au/Pd, Pt, Cr) under different coating times (1, 2, 4, 8, 16 min); The bright field images (**b**–**d**), AFM height images (**e**–**g**) and thickness curves (**h**) of metal films under the optimized coating times.

One of the key factors of nanofacula array is the shading property of metal films. Good shading coefficient can improve the contrast of the beam penetrating the nanopores, which is the guarantee to improve the energy of the beam. Therefore, we investigated the shading coefficients of Au/Pd, Pt and Cr under 1, 2, 4, 8, 16 min, respectively. Among them, the shading coefficient of Au/Pd film is the highest (99% at 8 min), while the Cr film is the lowest (98.6% at 16 min). It can be seen from the fitting curve shown in Fig. 3a, with the increase of coating time, the shading coefficient of the three kinds of metal films is obviously enhanced. Taking the shading coefficient of 90% as the standard, the optimized coating times of three metal films were 3.5, 5 and 8 min to Au/Pd, Pt and Cr, respectively. The dividing line between the metal film and the glass substrates can be clearly distinguished from the AFM bright field images (Fig. 3b–d). Using the AFM probe to get the height images (Fig. 3e–g) and the thickness curves (Fig. 3h) at the steps, the average thickness of the three metal films is Au/Pd 23.13 ± 5.32 nm, Pt 30.14 ± 3.89 nm and Cr 32.13 ± 2.54 nm, respectively (n > 30). As the lift-off process requires that the thickness of the PMMA is at least twice of the metal film, which directly affects the stability of the mushroom structure, so it is necessary to select the thinnest metal film with the highest shading coefficient. From the above data, it can be concluded that the coating time of 3.5 min Au/Pd film meets the preparation requirements of metal nanopore array.

### Thickness of PMMA and HSQ

**Figure 4:**
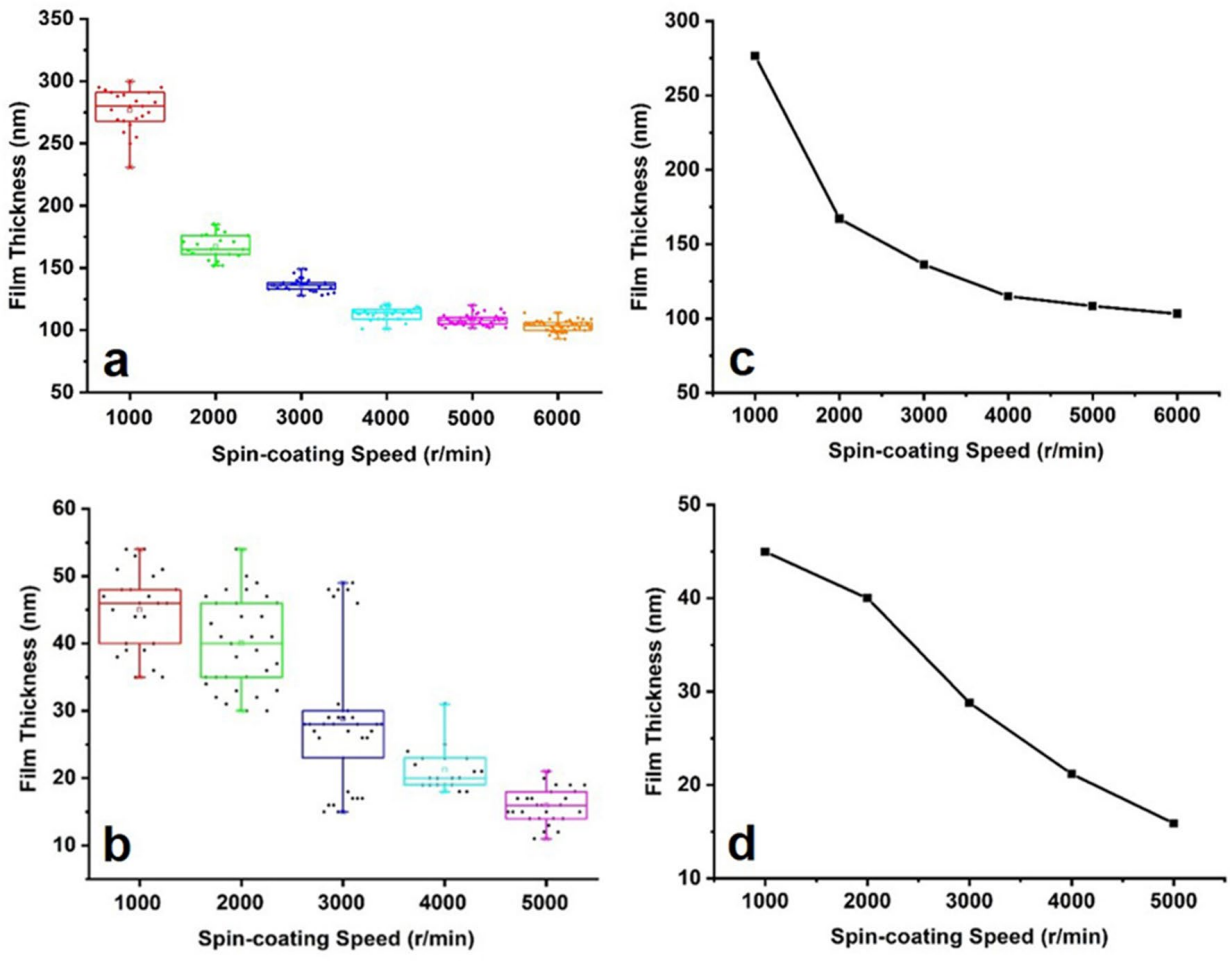
The box chart (**a**,**b**) and dot-line diagram (**c**,**d**) of PMMA and HSQ thickness at different spin-coating speeds. The area of the box in the box chart is 95% of all the data, and the horizontal line is the average thickness.

After determining the thickness of the metal film, the thickness of PMMA and HSQ was investigated at different spin-coating speeds. The box diagram (Fig. 4a,b) and average values, while the dot-line diagram (Fig. 4c,d) shows the trend that the resist thickness decreases with the increase of spin-coating speed, and the exact values. According to the optimized thickness of Au/Pd film, we choose the thickness of 130 nm corresponding to 3000 r/min as the spin-coating speed of PMMA in this experiment. For HSQ, a certain thickness is needed to ensure the stability of the capping of mushroom structure, so 2500 r/min corresponding to relatively stable 40 nm is selected as the spin-coating speed of HSQ in this experiment.

### Characterization of nanofacula array

**Figure 5:**
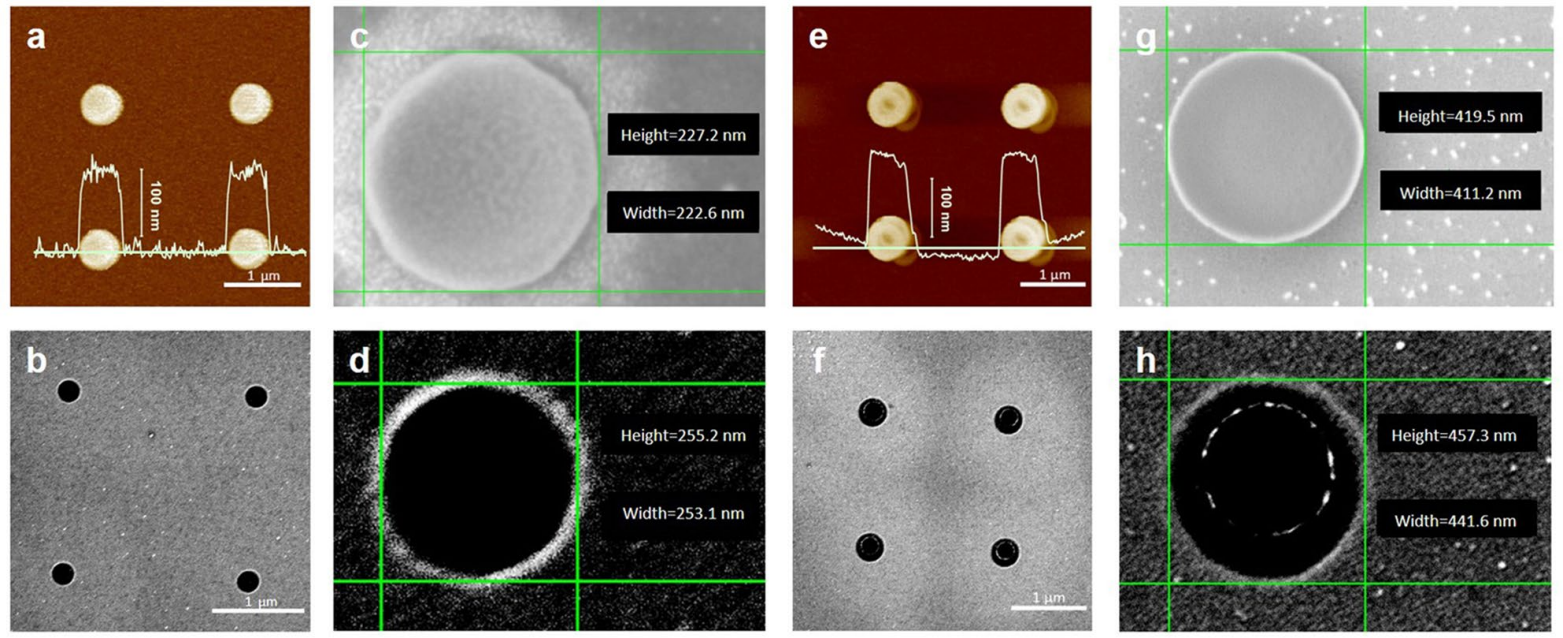
Characterization during the fabrication process of nanopore array. AFM topography of PMMA pillar and HSQ capping obtained by oxygen plasma etching (**a**,**e**) and the size measurement charts based on SEM (**c**,**g**). SEM photos of nanopore array after lift-off (**b**,**f**) and the size measurement charts based on SEM (**d**,**h**). The structures in (**a**–**d**) are 250 nm, and in (**e**–**h**) are 450 nm. The yellow curves are the height curves corresponding to the green lines in (**a**,**e**), and the scale bars in z-axis are 100 nm.

**Figure 6:**
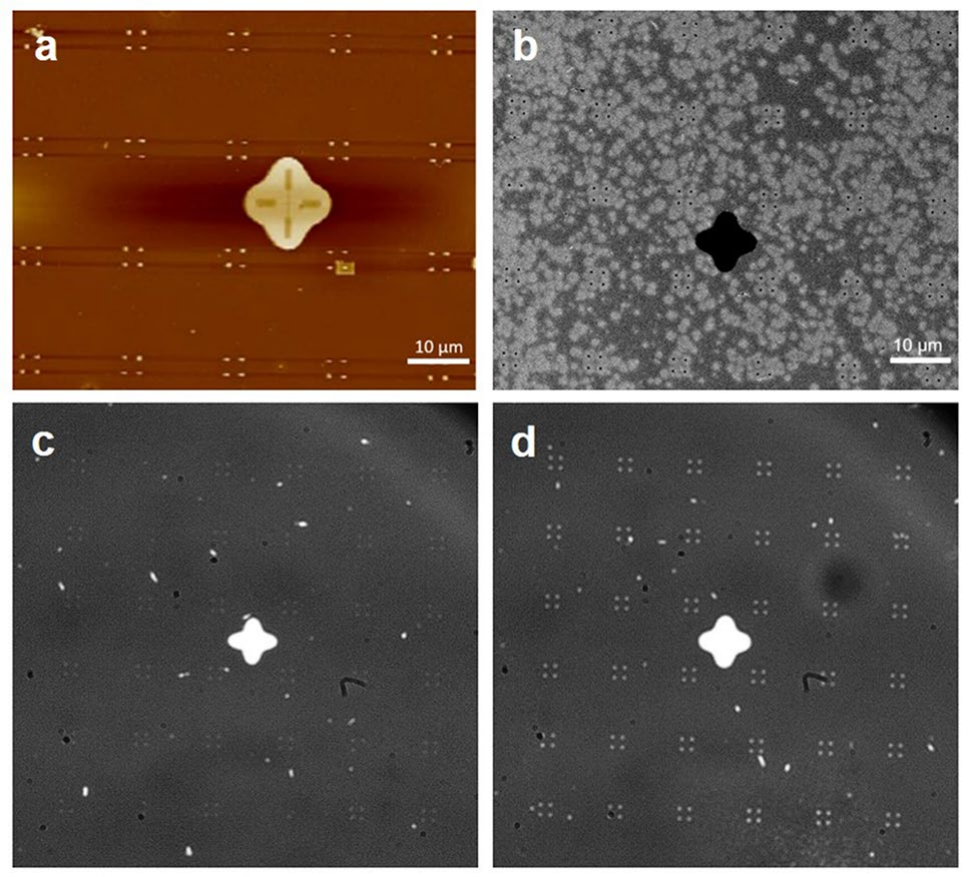
AFM topography of PMMA pillar and HSQ capping of the array obtained by oxygen plasma etching (**a**) and SEM photos of nanopore array after lift-off (**b**); Optically transparent nanofacula arrays with different apertures illuminated by white light (**c**,**d**). The structures in c are 250 nm, and in a, b and d are 450 nm.

Two kinds of array patterns are designed as pore spacing 2 μm, group spacing 7 μm, aperture 250 nm and 450 nm, respectively. After the etching of plasma, AFM images show that the height of the mushroom structures is between 130 and 170 nm (Fig. 5a,e), which is more than double the thickness of the metal film that is necessary for the subsequent lift-off process. Otherwise, the size of the HSQ capping measured by SEM are shown in Fig. 5c,g, the high precision of EBL and development was reflected by the clear edge of the circular capping and the small size error within 30 nm. SEM images of metal nanopore array after lift-off process (Fig. 5b,d,,f,h) displays the metal pores with well-designed shape, smooth edge and the small size error (less than 10 nm), which reflects the precise control of the parameters by this method. Figure 6 shows the AFM topography image (Fig. 6a), the SEM picture (Fig. 6b) of the nanopore array and the nanofacula array under white light irradiation (Fig. 6c,d). The successful realization of nanofacula array is benefit from the high completion and the good light transmission of metal nanopore array.

### Numerical analysis of nanopore-microlens array

**Figure 7:**
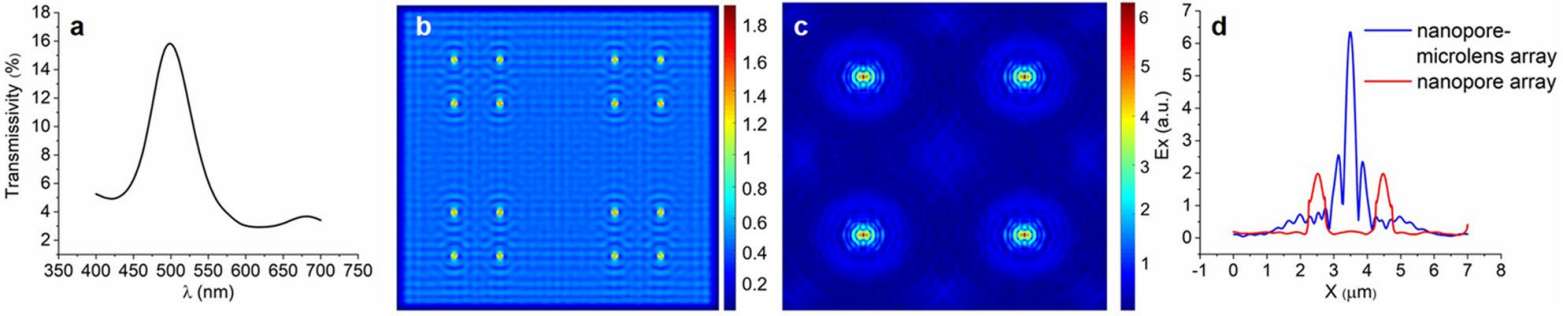
Transmission spectra curve (**a**); E-field distribution of nanopore array on x–y plane of z = 0 nm (**b**); E-field distribution of nanopore-microlens array on x–y plane of z = 20 nm (**c**); E-field profile of facula center (**d**).

Figure 7a shows the transmission spectrum of the nanopore array with the diameter of 250 nm, and it can be seen that there is a transmission peak value at 500 nm. Therefore, the incident wavelength of 500 nm and wavelength span of 20 nm is selected to detect the electric field (E-field) distribution of the nanopore array. Figure 7b shows the E-field distribution at Z = 0 nm away from the exit of the nanopores on x–y plane. The nanopore spot has an elliptical shape with size of 356 × 445 nm at the site of full width at half-maximum E-field amplitude. Due to the diffraction effect, the size of the facula is larger than that of the nanopore. In order to obtain smaller focused facula, the nanopore array structure is designed to combine with the microlens array, and each nanopore array unit corresponds to one microlens. Figure 7c shows the E-field distribution at Z = 20 nm away from the exit of the lens on x–y plane. As can be seen, the central-focused beam spot has an elliptical shape with size of 145 × 260 nm at the site of E-field amplitude, which is due to the linear polarization of the incident beam. The microlens radius, material and sample-probe distance are all optimized results. The sample-probe distance is given by the E-field distribution in the x–z plane and y–z plane, where appears with the smallest spot size. In Fig. 7d, the peak value of nanopore-mircolens array’s E-field is around 6.2, which shows that the E-field is enhanced 3.2 times in comparison to the nanopore array. The structure of the nanopore-mircolens array can achieve high throughput array scanning. It provides a new idea for NSOM to realize fast super-resolution focusing facula.

## Conclusion

Nano optical probe is one of the core part of NSOM, which determines the spatial and time resolution of NSOM through its size and quality. In this paper, we propose a method to improve the spatial resolution, which is using the structure of nanopore array combined with micolens array as the scanning probe. While constraining the incident light to the nanometer level, the nanofacula array develops single-spot scanning into array scanning, which can effectively improve the time resolution of NSOM. A method of preparing nanofacula array is introduced, which can accurately control the size, shape, spacing and other parameters of the facula array, and realize the array fabrication of large area (centimeter level). Moreover, the combination of nanopore array with micro-lens array is proved to realize super-resolution focused nanofacula array. Hence, this work provides a new way for NSOM to realize super-resolution focused nanofacula array.

## Supplementary Information

Supplementary Information.
