# Supplementary material for: Preparation and characteristic analysis of nanofacula array
Source: Sci Rep. 2021 Nov 12;11:22140. doi: 10.1038/s41598-021-01637-0 (PMC8590054; doi:10.1038/s41598-021-01637-0)
Supplement: Supplementary file 1 — Supplementary Information. [file 41598_2021_1637_MOESM1_ESM.docx]

**Supplementary information of “Preparation and Characteristic Analysis of Nanofacula Array”**

***Thickness of metal films using scratch method***

To eliminate the affection of the metal film when the tape was removed, and the adhesive residue remained on the junction of the film and tape, we measured the thickness of metal films using the scratch method, and compared the results. We used an iron knife to scratch the thin metal films of AuPb and Pt, and measure the thickness of the two metal films. Because the Mohs hardness of iron is higher than AuPb and Pt, but lower than Cr and glass, we can scratch the AuPb and Pt films without damage the glass substrate. But the Cr film cannot be scratched by iron knife, a glass cutter is not a suitable tool because of that it is very difficult to scratch only the film without the glass substrate damage. It may affect the accuracy of the thickness test, so we soak the Cr film in acetone after tape removed to clear away the adhesive residue, and measure the film thickness. Little difference existed between the average thickness of the three metal films, as Au/Pb 23.13±5.32 nm, Pt 30.14±3.89 nm and Cr 32.13±2.54 nm (tape method), while Au/Pb 26.85±5.46 nm, Pt 32.43±5.04 nm and Cr 42.15±4.12 nm (scratch method), respectively. The results obtained by the two methods are consistent with our choice of shading film.


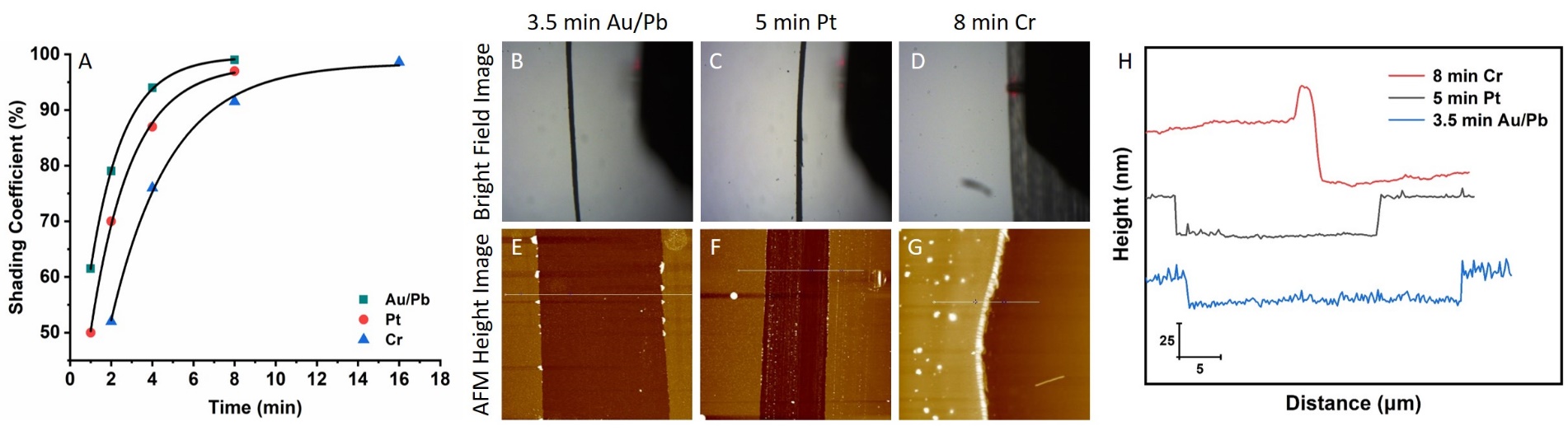


Fig. S1 (A) Shading coefficient of three kinds of metals (Au/Pb, Pt, Cr) under different coating times (1, 2, 4, 8, 16 min); The bright field images (B-D), AFM height images (E-G) and thickness curves (H) of metal films under the optimized coating times using scratch method.

***Preparation of nanofacula array***

1. Platinum (Pt) was sputtering on the clean glass surface for 15 s as a conductive layer.

2. Double layer resist coating: PMMA was spin coating with acceleration 6000 r/min to achieve the final rotational speed (3000 r/min), and the measured thickness is about 130 nm. Heat the PMMA in the glove box (160 ℃, 4 min). Then HSQ was spin coating with acceleration 6000 r/min to achieve the final rotational speed 2000 r/min, and the measured thickness is about 40 nm. Reheated for 90 s at 110 ℃ to prevent HSQ deformation caused by excessive temperature (>120 ℃).

3. EBL: the exposure voltage is set to 20 keV, the current is 50 pA, and the electron beam Dose is 4 mC/cm^2^. HSQ is exposed to form HSQ capping array.

4. Development and fixing: samples were soaked in 25%TMAH at room temperature for 80 s, then fixed in deionized water for 90 s and dry with nitrogen.

5. Oxygen plasma etching (plasma): set parameters as pressure of 0.36 pa, power of 100 W, flow coefficient of 35 sccm and time of 5 min to etch PMMA and obtain the mushroom structure of HSQ capping-PMMA column. In order to avoid the transverse size increase caused by the tip broadening effect of AFM, we use SEM to characterize the size of the HSQ capping and AFM to characterize the height of the mushroom structure.

6. Metal coating: set coating voltage E as 7 keV (6 keV for Cr), current I as 358 μA, coating time as 1, 2, 4, 8, 16 min with the three metals (Au/Pb, Pt, Cr).

7. Lift-off: the sample was ultrasonic 10 min twice in NMP solution, the PMMA was dissolved, the mushroom structure was destroyed, and the obtained metal nanopore array was characterized by SEM. If there is residual PMMA in the hole, it can be plasma again to ensure the light transmittance of the array.

***Numerical simulation process of FDTD Method***

1. Creating physical structures: the glass substrate was simulated by the rectangle in Structures tool, the substrate size was defined by X/Y/Z span parameters, which were set as x span=14 μm, y span=14 μm, Z span=0.6 μm, and the material of it was set as SiO_2_; a metal film was created by the same tool but with the Z span set as 0.06 μm, while the X/Y span set as 14 μm, the material of it was Au (gold)–CRC. 16 nanopores were established into the metal film using the circle tool, and the material was set with a radius of 125 nm. The center coordinates of 16 nanopores were x=±2.5, y=±2.5, x=±2.5, y=±4.5, x=±4.5, y=±2.5, x=±4.5, y=±4.5, y=±4.5.

2. Configuring simulation parameters: add a simulation area using the Simulation tool, and set the parameters of it as follows: dimension=3D，background index=1, simulation time (fs)=1000, x=y=z=0, x span=y span=16 μm, z span=3 μm, while mesh accuracy=2 or 3, minimum step=0.00025 μm were set in the mesh setting; PML boundary conditions can be selected in the boundary conditions. Due to the symmetry between the light source and the structure the boundary conditions can also be set as symmetric or anti-symmetric.

3. Defining radiation source: add the plane wave source with the Source tool, and set the parameters as follows: amplitude=1, phase (degrees)=0, projection axis=z-axis, direction=forward.

4. Defining monitor: add the monitor with the Monitor tool, and select the frequency domain field profile or frequency domain field and power detector to get the light field distribution and transmittance curves of a 2D-X/Y/Z plane.

5. Double-check: firstly, check the fitting parameters of material properties, select material explorer in the Check tool in the software, the fitting curves of the real part and imaginary part of the dielectric constant of gold material can be checked under fit and plot page. If the curves of the selected material were well-fitted in the band of 400-800nm, the selected material type was correct. Secondly, check the memory space required for simulation operation to ensure that the computer had enough resources to run the simulation. Select Check simulation and memory requirements under the Check tool to get the memory required for simulation calculation. If the required memory was too large and exceeds the configuration of the computer, it is necessary to consider altering the parameters to reduce the required memory.

6. Configuring the computer: when running the simulation for the first time, the computer needed configuration. Click Resources in the software and then click run tests to check whether the test was successful. If the configuration test passed, success appears in the status column below. After successful configuration, click Save to save.

7. Running simulation and extracting data: click the Run button to start the simulation. Select monitor in the software and the simulation data will be displayed in the result view. Right click E under the result view and select new visualizer to get the electric field distribution map.

8. Adding microlens array: add a hemisphere in Components in step 1, set the parameters as follows: index=1.51, radius=3.5 μm, four micro-lenses whose center coordinates were created as x=±3.5, y=±3.5. Repeat steps 2-7 to obtain the electric field distribution map of the radiation light passing through the nanopore microlens array structure.
